# Identification of volatile metabolites produced from levodopa metabolism by different bacteria strains of the gut microbiome

**DOI:** 10.1186/s12866-024-03373-7

**Published:** 2024-07-13

**Authors:** Taylor Pennington, Jarrett Eshima, Barbara S. Smith

**Affiliations:** School of Biological and Health Systems Engineering, Arizona State University, Tempe, AZ 85287 USA

**Keywords:** Volatile Organoid Compound, Levodopa, Gut microbiota, Metabolism

## Abstract

Interspecies pathways in the gut microbiome have been shown to metabolize levodopa, the primary treatment for Parkinson’s disease, and reduce its bioavailability. While the enzymatic reactions have been identified, the ability to establish the resulting macromolecules as biomarkers of microbial metabolism remains technically challenging. In this study, we leveraged an untargeted mass spectrometry-based approach to investigate volatile organic compounds (VOCs) produced during levodopa metabolism by *Enterococcus faecalis*, *Clostridium sporogenes*, and *Eggerthella lenta*. We cultured these organisms with and without their respective bioactive metabolites and detected levodopa-induced shifts in VOC profiles. We then utilized bioinformatics to identify significant differences in 2,6-dimethylpyrazine, 4,6-dimethylpyrimidine, and 4,5-dimethylpyrimidine associated with its biotransformation. Supplementing cultures with inhibitors of levodopa-metabolizing enzymes revealed specific modulation of levodopa-associated diazines, verifying their relationship to its metabolism. Furthermore, functional group analysis depicts strain-specific VOC profiles that reflect interspecies differences in metabolic activity that can be leveraged to assess microbiome functionality in individual patients. Collectively, this work identifies previously uncharacterized metabolites of microbe-mediated levodopa metabolism to determine potential indicators of this activity and further elucidate the metabolic capabilities of different gut bacteria.

**Supplementary Information:**

The online version contains supplementary material available at 10.1186/s12866-024-03373-7.

## Introduction

Parkinson’s disease (PD) is a neurodegenerative disorder that predominantly affects dopamine-producing neurons in the brain, resulting in hallmark symptoms including tremors, rigidity, slowed movements, and impaired balance [1]. The primary treatment for PD is levodopa, an orally ingested central nervous system agent that is metabolized by aromatic amino acid decarboxylase (AADC) enzymes in the brain to produce dopamine [2]. First-pass metabolism outside the nervous system heavily impairs therapeutic efficacy, since dopamine generated in the periphery cannot cross the blood-brain barrier [3]. However, despite its co-prescription with peripheral AADC inhibitors, patients display highly variable treatment responses and reduced efficacy over time. Recent studies have identified bacteria strains in the gut microbiome as major factors that reduce levodopa bioavailability [4, 5]. Previous work from Rekdal et al. established an interspecies pathway that begins with the bioconversion of levodopa to dopamine by *Enterococcus faecalis*, followed by dopamine dehydroxylation to *m*-tyramine by *Eggerthella lenta* [4]. In an alternative pathway, the prevalent gut microbe *Clostridium sporogenes* converts levodopa to 3-(3,4-dihydroxyphenyl) propionic acid (DHPPA), which is further degraded into 3-(3-hydroxyphenyl) propionic acid (3-HPPA) by *Eggerthella lenta* [5]. While previous efforts have sought to directly detect levodopa metabolites from biological samples [6–8], there remains an inability to establish these macromolecules as reliable indicators of extracerebral metabolism due to discrepancies in sampling and analytical techniques. The lack of standardized sampling procedures, coupled with differences in the specificity and sensitivity of analytical instruments, introduces consequential variability in metabolite measurements that create difficulty deriving biological interpretations from results [9–12]. Such challenges underscore the need for a more robust method to evaluate levodopa metabolism in Parkinson’s patients and assess interindividual variations in treatment response.

Metabolomics has been identified as a powerful tool to enhance our understanding of the metabolic repertoire of gut bacteria and the discovery of microbiome-drug interactions. Microbes have a distinct metabolism and produce an array of metabolites, including bacteria-specific volatile organic compounds (VOCs) that can cross the blood-brain barrier and enter host circulation [13–15]. VOCs are small molecules that represent the volatile portion of the metabolome and have gained attraction as a non-invasive method for diagnosis and assessing treatment efficacy [14–17]. In contrast to macromolecule-based biomarkers, VOCs can be sampled non-invasively and with minimal preparation, providing a direct detection method with potential for translational research. Recent studies have demonstrated that VOCs characterized from biological mediums can reflect microbiome composition and distinguish physiological states, essential in understanding the mechanisms underlying host-microbiome interactions [14, 15, 18, 19]. Facilitating the analysis of VOCs, gas chromatography-mass spectrometry (GC-MS) remains the gold standard for the untargeted analysis of VOCs extracted from biological systems [13, 15]. Direct extraction of volatile metabolites from the headspace above microbial cultures provides a non-destructive means to assess metabolic activity and improves the recovery of low abundant compounds for untargeted analysis with GC-MS. As a result, there are a growing number of studies using GC-MS to analyze the composition and chemical reaction space of the human microbiome. Expanding the scope of gut microbial-related metabolites enables the identification of diverse microbiome-drug interactions that can differentiate metabolic phenotypes. Consequently, volatile signatures of levodopa metabolism represent a potential strategy to directly evaluate heterogeneous responses and account for the microbiome’s effect on pharmacokinetics.

This study builds off foundational works to identify previously uncharacterized metabolites from bacteria strains involved in levodopa metabolism to better understand the biological significance of this activity. We employed an untargeted approach with GC-MS to profile VOCs from *Enterococcus faecalis* strain OG1RF, *Clostridium sporogones* (WT), and *Eggerthella lenta* strain MSMC 77 − 67 (DSM 15644) and identify discriminant compounds of levodopa metabolism. Statistical analysis comparing cultures with and without levodopa enabled the identification of VOCs uniquely produced during the breakdown of the drug. Inhibiting drug-microbe interactions with compounds that inactivated the corresponding enzymes resulted in the modulation of levodopa-related VOCs, further demonstrating their association with its metabolism. These results identify previously unknown metabolites of bacterial levodopa metabolism that correlate with metabolic pathways involved in cell growth, offering additional insights into the cellular mechanisms impacting pharmacology. Furthermore, strain-specific VOCs corresponding to each pathway offer a robust strategy to inform personalized treatment strategies based on interpersonal microbiome compositions.

## Results

### *Enterococcus faecalis* produces diazine by-products during levodopa decarboxylation

**Fig. 1:**
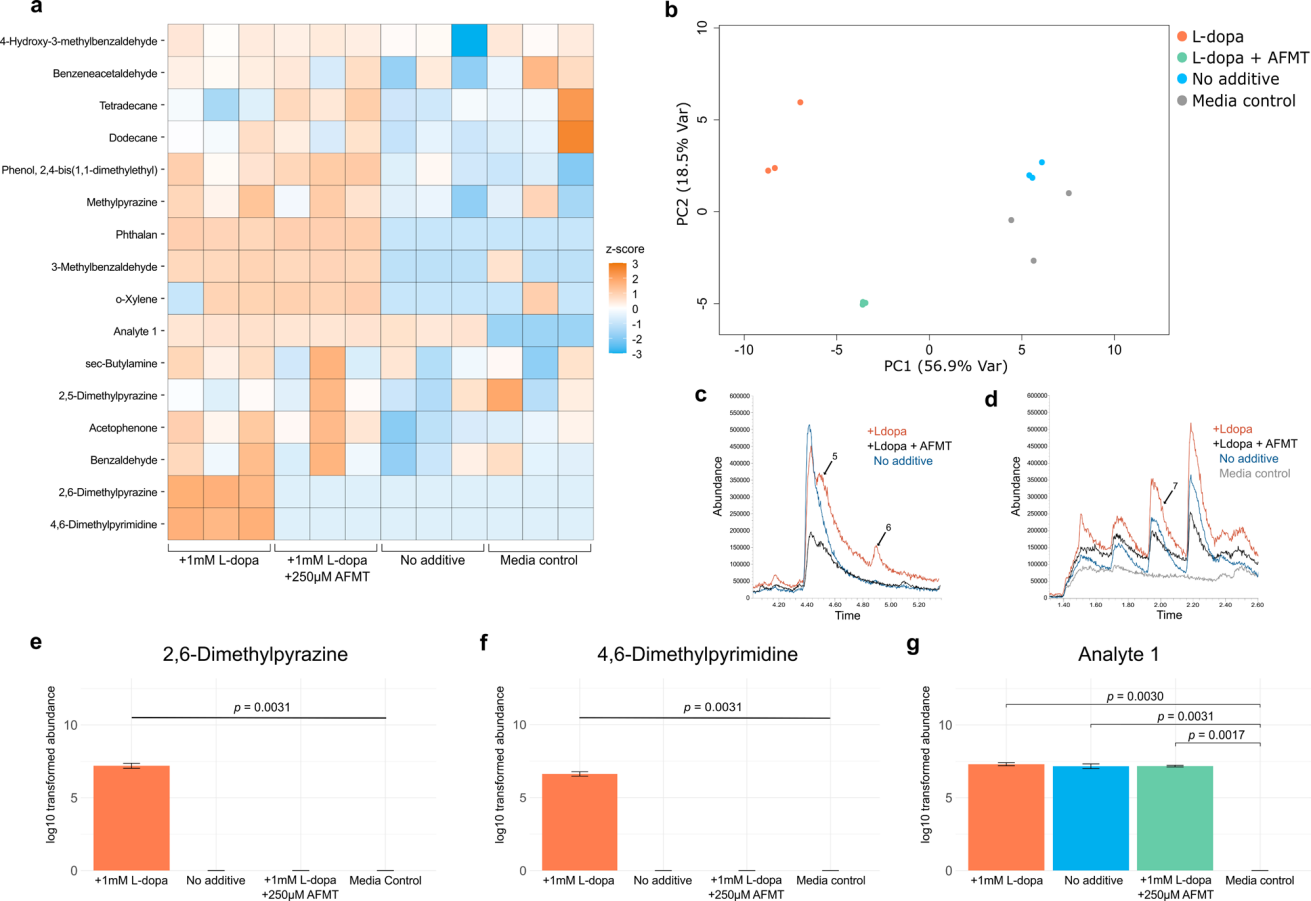
VOCs produced during levodopa decarboxylation by *E. faecalis*: **a**. Heatmap of volatile metabolites detected from cultures of *E. faecalis* shows changes in VOC abundance across each experimental condition. **b**. Principal component analysis shows four distinct clusters of VOC profiles when considering the first two principal components. **c, d**. Representative chromatograms from each experimental condition and labeled peaks corresponding to statistically significant compounds. **e**-**g**. Log transformed abundance of significant VOCs detected from *E. faecalis* and corresponding FDR-adjusted *p*-values

To collect VOCs from bacteria cultures, strains were inoculated into a custom culture vessel previously developed in our lab to enable the continuous flow of gas through the headspace [20], effectively increasing overall sensitivity and reproducibility of collected VOCs (Fig. S1). In short, gas is designed to flow continuously from the vessel inlet to the outlet and continuously deplete analytes from the headspace, increasing the total available signal as compared to static headspace sampling methods. To investigate differences in VOC production during the bioconversion of levodopa to dopamine, we collected VOCs from the strain *E. faecalis* OG1RF cultured with and without 1mM levodopa. VOCs were recovered using solid-phase microextraction (SPME) and characterized by GC-MS analysis. Sixteen VOCs spanning a range of chemical classes were recovered from *E. faecalis* (Table 1). Comparison of volatile metabolomes from each experimental replicate shows differential VOC abundance in *E. faecalis* cultures containing levodopa (Fig. 1a). The chromatograms generated from each experimentalgroup depict compounds endogenously produced from E. faecalis (VOCs not detected in the growth media) and compounds uniquely produced during levodopa decarboxylation (Fig. 1c, Fig. 1d, Fig. 1g). Statistical analysis performed in R identified significant changes in 2,6-dimethylpyrazine and 4,6-dimethylpyrimidine (adjusted p < 0.05) during levodopa decarboxylation and a significant difference in Analyte 1 from E. faecalis replicates and the media control (Fig. 1e, Fig. 1f, Table S1). Applying a multiple hypothesis test correction21 with a false discovery rate (FDR) set at 0.10 maintained significant differences. Chromatographic peaks corresponding to 2,6-dimethylpyrazine, 4,6-dimethylpyrimidine, and Analyte 1 are indicated by arrows and the corresponding number for which they are listed in Table 1.

**Table 1:** Filtered VOC list from E. faecalis OG1RF

Number	VOC name	Chemical Class	Retention Time (min)	RI	ID Level
1	sec-Butylamine	Amine	1.95	469	3
2	Analyte 1	Unknown	2.23	478	4
3	Methylpyrazine	Heteroaromatic	3.21	828	2
4	o-Xylene	Aromatic	4.18	901	2
5	2,5-Dimethylpyrazine	Heteroaromatic	4.42	914	1
**6**	**2,6-Dimethylpyrazine**	**Heteroaromatic**	**4.5**	**918**	**1**
**7**	**4,6-Dimethylpyrimidine**	**Heteroaromatic**	**4.89**	**938**	**2**
8	Benzaldehyde	Aldehyde	5.4	964	2
9	Benzeneacetaldehyde	Aldehyde	7.22	1048	2
10	Acetophenone	Ketone	7.77	1071	2
11	4-Hydroxy-3-methylbenzaldehyde	Other	8.03	1082	3
12	3-Methylbenzaldehyde	Aldehyde	8.14	1087	2
13	Phthalan	Other	8.58	1108	3
14	Dodecane	Hydrocarbon	9.83	1200	1
15	Tetradecane	Hydrocarbon	11.36	1399	1
16	Phenol, 2,4-bis(1,1-dimethylethyl)	Other	11.99	1522	2

List of identified VOCs from *E. faecalis* cultures and their chemical classifications. “Other” is defined as >1 functional group. VOCs identified as significant to levodopa metabolism are shown in bold. The retention index (RI) was calculated based on each compound’s retention time using KI standards. ID level is displayed in the final column

The foundational work from Rekdal et al. shows that the L-tyrosine analog, (S)-α-fluoromethyltyrosine (AFMT), effectively prevents levodopa decarboxylation in *E. faecalis* cultures by selectively inhibiting the tyrDC enzyme [4]. Therefore, we hypothesized that supplementing cultures of *E. faecalis* with levodopa in combination with AFMT would inhibit the production of 2,6-dimethylpyrazine and 4,6-dimethylpyrimidine. We found that adding AFMT (250 µM) generated similar VOC profiles to *E. faecalis* cultures without levodopa. The abundance of 2,6-dimethylpyrazine and 4,6-dimethylpyrimidine were significantly inhibited following coadministration of AFMT and levodopa. Principal component analysis (PCA) demonstrated the ability to separate the VOCs detected across all four experimental conditions into distinct clusters when considering the first and second principles, although it is evident that the native bacterial volatile profile overlaps with broth volatile profile – as is commonly reported [20–22] (Fig. 1b). Together, these findings report volatile diazines produced by *E. faecalis* in the presence of levodopa that are eradicated when its biotransformation is inhibited with AFMT. These results suggest the production of 2,6-dimethylpyrazine and 4,6-dimethylpyrimidine to be potential indicators of levodopa decarboxylation.

### Increased production of fatty acid esters observed during levodopa deamination by *Clostridium sporogenes*

To investigate each pathway for gut bacterial levodopa metabolism, we next worked to determine whether *C. sporogenes* produced VOCs associated with levodopa deamination. Using the same methods described previously, we detected VOCs from cultures of *C. sporogenes* with and without levodopa to capture differences in VOC production during the biotransformation to DHPPA (Table 2). The list of VOCs detected from *C. sporogenes* contained 41 compounds spanning a diverse range of chemical classes.

**Table 2:** Filtered VOC list from C. sporogenes

Number	VOC name	Chemical Class	Retention Time (min)	RI	ID Level
1	Analyte 1	Unknown	1.50	453	4
2	Analyte 2	Unknown	1.53	454	4
3	Analyte 3	Unknown	1.67	460	4
4	Analyte 4	Unknown	1.71	465	4
5	Analyte 5	Unknown	1.85	468	4
6	sec-Butylamine	Amine	1.95	469	3
7	Analyte 6	Unknown	2.34	482	4
8	Analyte 7	Unknown	2.63	493	4
9	4-Methyl-1-pentanol	Alcohol	3.26	832	3
10	2,5-Dimethylpyrazine	Heteroaromatic	4.42	914	1
11	Benzaldehyde	Aldehyde	5.4	964	2
12	Butanoic acid, butyl ester	Ester	6.03	996	2
13	Trimethylpyrazine	Heteroaromatic	6.2	1004	3
14	Butyl 2-methylbutanoate	Ester	7.09	1042	2
15	Butanoic acid, 3-methyl-, butyl ester	Ester	7.19	1046	2
16	Pentanoic acid, butyl ester	Ester	7.43	1056	3
17	Acetophenone	Other	7.77	1071	2
18	Butanoic acid, pentyl ester	Ester	8.32	1094	3
19	Butanoic acid, 2-methyl-, 3-methylbutyl ester	Ester	8.46	1100	2
20	Butanoic acid, 3-methyl-, 3-methylbutyl ester	Ester	8.55	1107	2
21	n-Amyl isovalerate	Ester	8.61	1111	2
22	Phenylethyl alcohol	Alcohol	8.74	1120	2
23	Benzyl nitrile	Nitrile	9.16	1151	3
24	Hexanoic acid, 3-methylpropyl ester	Ester	9.33	1163	3
25	Dodecane	Hydrocarbon	9.84	1200	1
26	Hexyl n-valerate	Ester	9.89	1206	3
27	Isopentyl hexanoate	Ester	10	1219	2
28	Benzenepropanol	Alcohol	10.18	1239	2
29	Hexanoic acid, pentyl ester	Ester	10.33	1257	3
30	Analyte 8	Unknown	10.82	1317	4
31	Analyte 9	Unknown	11.07	1355	4
32	Tetradecane	Hydrocarbon	11.36	1399	1
33	Phenethyl butyrate	Ester	11.6	1445	3
34	Benzeneacetic acid, butyl ester	Ester	11.63	1450	2
35	Butanoic acid, 3-methyl-, 2-phenylethyl ester	Ester	11.91	1504	3
36	Phenol, 2-4-bis(1,1-dimethylethyl)	Other	11.99	1522	2
37	Analyte 10	Unknown	12.10	1545	4
38	2-Methylpropyl benzenepropanoate	Ester	12.17	1560	3
39	Butanoic acid, 3-phenyl propyl ester	Ester	12.22	1573	2
40	Hexadecane	Hydrocarbon	12.35	1599	1
41	Pivalic acid, 2-phenylethyl ester	Ester	12.44	1621	3

List of identified VOCs from *C.sporogenes* cultures and their chemical classifications. “Other” is defined as >1 functional group. The retention index (RI) was calculated based on each compound’s retention time using KI standards. ID level is displayed in the final column

**Fig. 2:**
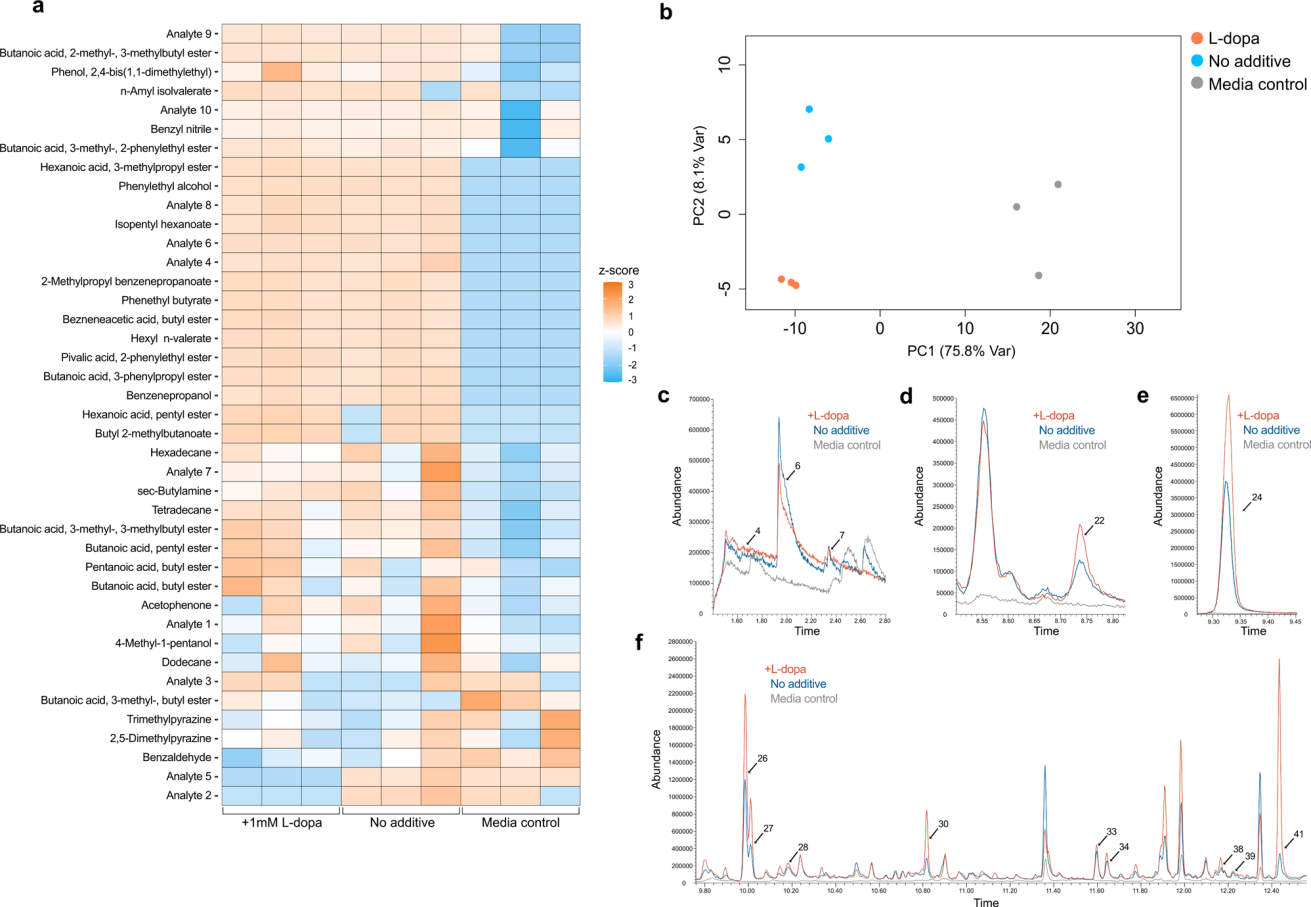
Characterization of VOC profiles generated from *C. sporogenes*: **a**. Heatmap of volatile metabolites detected from cultures of *C. sporogenes* shows changes in VOC abundance across each experimental condition. **b**. Principal component analysis shows three distinct clusters of VOC profiles when considering the first two principal components, with distinct VOC profiles generated with and without the presence of levodopa. **c-f**. Representative chromatograms from each experimental condition and labeled peaks corresponding to endogenous VOCs produced from *C. sporogenes*

GC-MS analysis revealed changes in VOC profiles acquired from *C. sporogenes* across each experimental condition (Fig. 2a). Furthermore, PCA shows three distinct clusters of VOC profiles corresponding to each experimental condition and reflects levodopa-induced shifts in VOC abundance (Fig. 2b). Statistical analysis was performed to further investigate the observed differences in VOC profiles between each condition. The statistical results did not reveal VOCs significant to levodopa deamination. While significant differences in Analyte 2 and Analyte 5 were detected between *C. sporogenes* cultured with and without levodopa, no significant differences were seen between levodopa replicates and the media controls, indicating that additional work is needed to determine possible metabolic origins. Although no VOCs were exclusively produced in the presence of levodopa, we detected increased compound abundances and overall shifts in volatile profiles corresponding to levodopa deamination.

Additionally, we identified significant differences between VOC profiles across *C. sporogenes* replicates and media controls, with adjusted *p*-values < 0.05 (Table S2). These results indicate the endogenous production of VOCs significant to *C. sporogenes* cultures and are depicted in the chromatograms from each condition (Fig. 2c-f). Chromatographic peaks corresponding to endogenously produced VOCs are indicated by arrows with the corresponding number for which they are listed in Table 2. Endogenous VOCs identified from *C. sporogenes* were mainly classified as esters.

### Volatile pyrimidine indicators of *Eggethella lenta* dehydroxylase activity generated during the degradation of levodopa metabolites

**Fig. 3:**
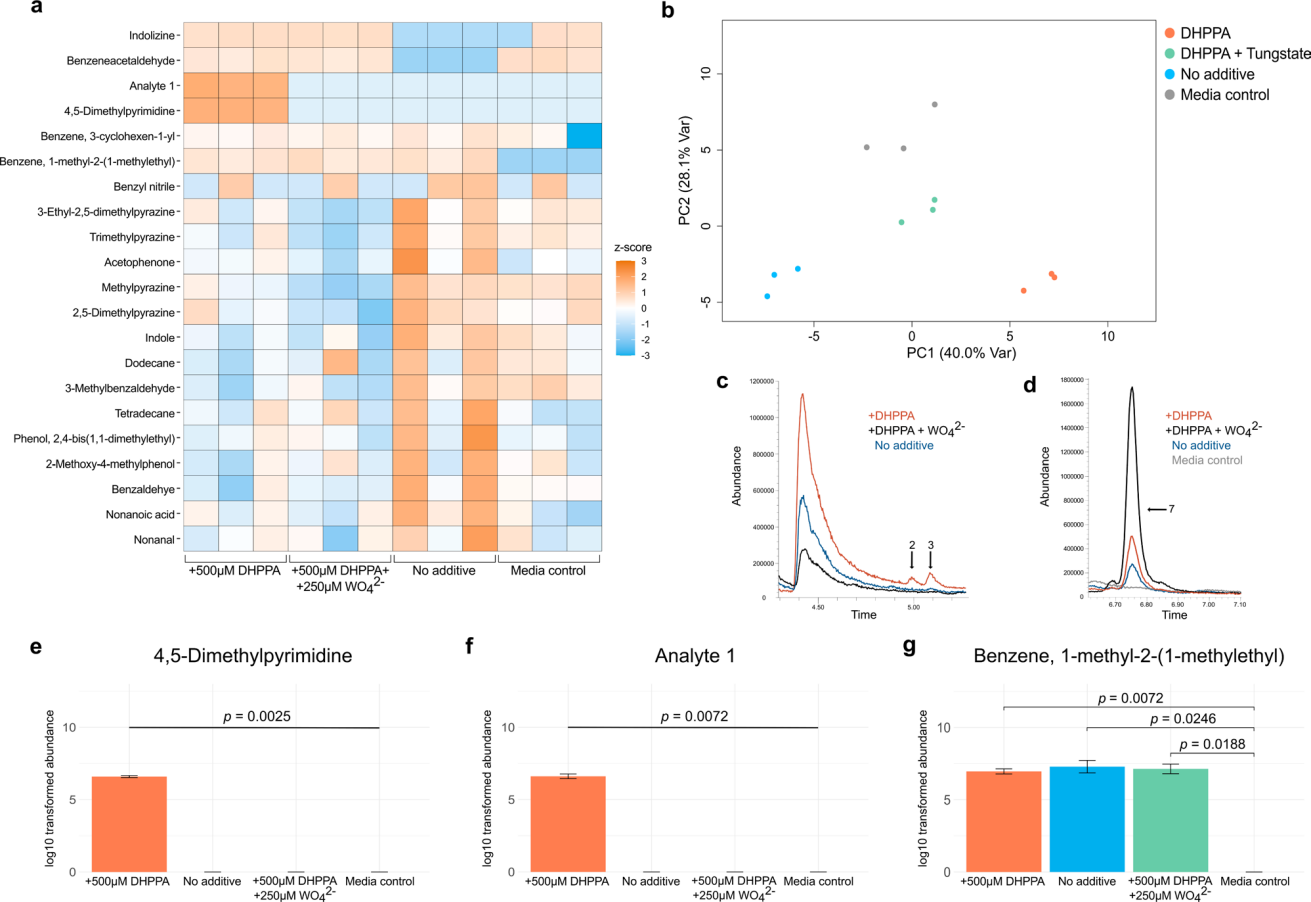
VOCs produced during DHPPA dehydroxylation by *E. lenta*: **a**. Heatmap of volatile metabolites detected from cultures of *E. lenta* shows changes in VOC abundance across each experimental condition. **b**. Principal component analysis shows four distinct clusters of VOC profiles when considering the first two principal components. **c, d**. Representative chromatograms from each experimental condition and labeled peaks corresponding to statistically significant compounds. **e**-**g**. Log transformed abundance of significant VOCs detected from *E. lenta* and corresponding FDR-adjusted *p*-values

The resulting intermediates from microbial levodopa metabolism, dopamine and DHPPA, are further metabolized by *E. lenta* via catechol dehydroxylase enzymes. Dopamine (dadh) and hydrocaffeic acid (hcdh) are the most prevalent catechol dehydroxylase enzymes that specifically metabolize the levodopa intermediates dopamine and DHPPA, respectively [23]. However, since a single nucleotide polymorphism (SNP) in the *dadh* gene distinguishes metabolizing and non-metabolizing *E. lenta* strains, we chose to investigate VOCs produced during DHPPA dehydroxylation to 3-HPPA by hcdh. The resulting VOCs exhibit differential abundances in *E. lenta* DSM 15,644 cultured with and without DHPPA (Fig. 3a). The list of VOCs reported for *E. lenta* contained 21 compounds, with the main chemical class being heteroaromatic (Table 3). Culturing *E. lenta* with 500 µM DHPPA resulted in a significant increase of 4,5-dimethylpyrimidine and an unknown compound labeled Analyte 1 (Fig. 3c-f). Further, benzene, 1-methyl-2-(1-methylethyl) was generated in all conditions except for the media control, confirming its endogenous origins to *E. lenta* metabolism (Fig. 3g). Chromatographic peaks for each VOC show changes in abundance across experimental conditions, and are depicted with arrows and the corresponding number which they are listed in Table 3. The unidentified compound labeled Analyte 1 has a similar retention index and the same parent peak as the other diazine compounds identified in this study (m/z = 102), likely indicating that the unidentified compound is a dimethylpyrimidine isomer.

To confirm that the dimethylpyrimidine compounds were a result of DHPPA dehydroxylation by hcdh enzymes, we supplemented cultures of *E. lenta* with sodium tungstate in combination with DHPPA. The inclusion of tungstate inactivates dehydroxylase enzymes without hindering bacteria growth by substituting the necessary molybdenum cofactor required for their activity [4]. We hypothesized that inhibiting the activation of dadh by supplementing *E. lenta* cultures with tungstate would yield decreased abundances of dimethylpyrimidines detected during DHPPA dehydroxylation.

As a result, supplementing 250 µM tungstate to *E. lenta* cultures in combination with 500 µM DHPPA significantly decreased the concentration of 4,5-dimethylpyrimdine and its putative isomer (Table S3). The inhibition of 4,5-dimethylpyrimidine and its putative isomer indicates the relationship of these VOCs with DHPPA dehydroxylation. Furthermore, each experimental condition produced characteristic VOC profiles that can be separated into distinct clusters when considering the first and second principal components (Fig. 3b). Inhibiting the dehydroxylase enzymes with tungstate yielded a more similar VOC profile to the media control compared to *E. lenta* cultured with no additives, possibly indicating off-target tungstate inhibition of the other molybdenum-dependent enzymes. Collectively, these findings identify dimethylpyrimidine VOCs significant to *E. lenta* cultured with DHPPA and also report the endogenous production of benzene, 1-methyl-2-(1-methylethyl). The inhibition of DHPPA-associated VOCs with tungstate suggests that increased dehydroxylase activity during the bioconversion to 3-HPPA shifts the metabolic activities of *E. lenta* that can be depicted in the volatile metabolome of this organism. 

**Table 3:** Filtered VOC list from E. lenta ATCC 43,055

Number	VOC name	Chemical Class	Retention Time (min)	RI	ID Level
1	Methylpyrazine	Heteroaromatic	3.21	828	2
2	2,5-Dimethylpyrazine	Heteroaromatic	4.41	913	1
**3**	**4,5-Dimethylpyrimidine**	**Heteroaromatic**	**4.89**	**938**	**3**
**4**	**Analyte 1**	**Unknown**	**5.09**	**948**	**3**
5	Benzaldehyde	Aldehyde	5.4	964	2
6	Trimethylpyrazine	Heteroaromatic	6.2	1004	3
7	Benzene, 1-methyl-2-(1-methylethyl)	Aromatic	6.75	1028	2
8	Benzeneacetaldehyde	Aldehyde	7.22	1047	2
9	Acetophenone	Other	7.77	1071	2
10	3-Ethyl-2,5-dimethylpyrazine	Heteroaromatic	8.02	1082	2
11	3-Methylbenzaldehyde	Aldehyde	8.14	1087	2
12	Nonanal	Aldehyde	8.54	1106	2
13	Benzyl nitrile	Nitrile	9.15	1150	2
14	Dodecane	Hydrocarbon	9.83	1200	1
15	2-Methoxy-4-methylphenol	Other	9.9	1208	2
16	Nonanoic acid	Acid	10.48	1274	2
17	Indole	Heteroaromatic	10.75	1306	2
18	Indolizine	Heteroaromatic	10.78	1311	3
19	Benzene, 3-cyclohexen-1-yl	Aromatic	11.19	1373	3
20	Tetradecane	Hydrocarbon	11.36	1399	1
21	Phenol, 2-4-bis(1,1-dimethylethyl)	Other	11.99	1522	2

List of identified VOCs from *E. lenta* cultures and their chemical classifications. “Other” is defined as >1 functional group. VOCs identified as significant to DHPPA metabolism are shown in bold. The retention index (RI) was calculated based on each compound’s retention time using KI standards. ID level is displayed in the final column

### Functional group analysis identifies strain-specific VOC profiles that reflect differences in metabolism

**Fig. 4:**
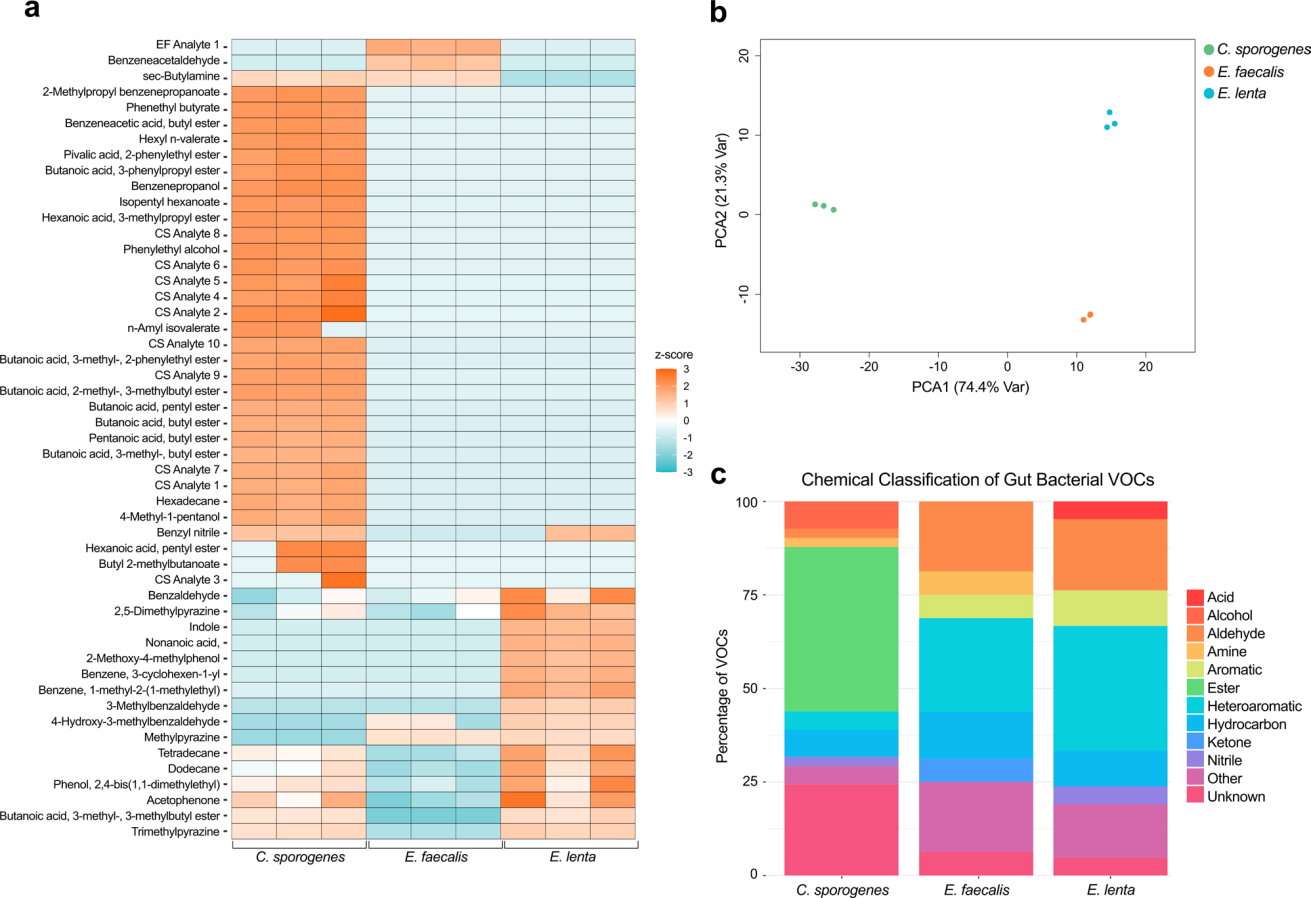
Chemical characterization of strain-specific VOC profiles: **a.** Heatmap showing differences in VOC abundance across *C. sporogenes*, *E. faecalis*, and *E. lenta*. **b.** PCA shows three distinct clusters of VOC profiles corresponding to each strain. **c.** Functional group analyses displays different proportions of VOCs based on the chemical classification

To capture unique metabolic activities harnessed by microbiota involved in levodopa metabolism, we analyzed the chemical diversity of each strain’s VOC profile. To do so, we compared VOCs detected across all three strains and analyze differential VOC abundance and frequencies(Fig. 4a). VOCs detected across all three strains include 2,5-dimethylpyrazine, benzaldehyde, acetophenone, dodecane, tetradecane, and phenol, 2,4-bis(1,1-dimethylethyl). Aside from these shared VOCs, the results from PCA yielded clusters of VOC profiles captured from *E. faecalis*, *C. sporogenes*, and *E. lenta* that showed highly distinct separation by strain (Fig. 4b). To better assess organism-specific volatile signatures, compounds were classified by functional group to further characterize the underlying biochemical behaviors. Functional group breakdown revealed unique proportions and categories of metabolites detected from each strain (Fig. 4c). The volatile metabolome of *C. sporogenes* was dominated by esters (43.90%), followed by unknown compounds (24.39%). Heteroaromatic compounds were the most prevalent VOCs detected from *E. faecalis* (25%), followed by aldehydes and VOCs classified as “other” (18.75% each). The most frequent chemical class detected from *E. lenta* was heteroaromatic VOCs (33.33%), followed by aldehydes (19.05%). Collectively, these results show VOCs of different chemical classes produced from *C. sporogenes*, *E. faecalis*, and *E. lenta*, and further highlight the distinct metabolic functions of different bacteria strains that exist in the gut.

## Discussion

In this study, we use an untargeted mass spectrometry-based approach to characterize VOCs from *E. faecalis*, *C. sporogenes*, and *E. lenta* and identify previously uncharacterized metabolites of bacterial levodopa metabolism. Many human microbiome studies seeking to understand the metabolic capacity of complex communities have highlighted the need to elucidate the molecular mechanisms contributing to the gut’s biochemical diversity [14–17]. Such efforts have prompted more targeted analyses of microbial metabolism to characterize the pathways encoded by individual strains and determine their biological significance. The discovery of gut microbial-derived VOCs has become a growing area of research that can provide major mechanistic insights into the biological processes underlying host-microbiome interactions with applications for personalized medicine. The main advantage of identifying characteristic VOCs over traditional biomarkers of drug metabolism is the reduction of analytical complexity. While the presence of levodopa-metabolizing enzymes can gauge potential gut microbial activities, the high implementation costs to extract and analyze these markers limits their routine utilization as indicators of off-target metabolism. Hence, VOC signatures of levodopa metabolism offer a robust, microbiome-centered approach to assess individual changes in its pharmacokinetics.

Studies have demonstrated a positive correlation between *tyrDC* gene expression, substrate concentration, and growth of *E. faecalis*, indicating that higher tyrDC activity enhances bacterial growth [24–27]. Importantly, the VOCs associated with levodopa decarboxylation correspond to metabolic activities tied to cell growth. These effects are further supported by functional analyses at the transcriptional level, which reveal that an increased tyrDC substrate concentration leads to enhanced pathways for purine and pyrimidine catabolism for DNA biosynthesis, along with an activation of amino-sugars metabolism in *E. faecalis* [27]. Furthermore, the accumulation of reactive α-hydroxycarbonyl and α-aminocarbonyl intermediates during the growth phase of various bacteria has been correlated with an increased production of pyrazine VOCs, which are widely associated with lactic acid bacteria [28–32]. These findings suggest that biological pathways corresponding to energy metabolism and growth may potentially give rise to the increased production of 2,6-dimethylpyrazine and 4,6-dimethylpyrimidine. The presence of these levodopa-induced diazines could potentially indicate a shift in *E. faecalis* metabolic activity in response to increased tyrDC substrate abundance. Notably, the results show that inhibiting levodopa decarboxylation with AFMT prevents the production of 2,6-dimethylpyrazine and 4,6-dimethylpyrimidine. The simultaneous inhibition of these compounds with AFMT suggests that these VOCs indicate a shift in *E. faecalis* metabolic activity in response to levodopa treatment and increased activity towards tyrDC substrates. Taking into account the low concentration of 2,6-dimethylpyrazine and 4,6-dimethylpyrimidine produced during levodopa decarboxylation by *E. faecalis*, follow-up analyses using complimentary methods with increased resolution and sensitivity, such as proton transfer traction mass spectrometry (PTR-MS) or selected ion flow tube mass spectrometry (SIFT-MS), would entail the selective detection and accurate quantification of these VOCs [33–35]. Parallel applications of such methods for quantitative determination of these compounds from complex microbiota samples would further their utility to potentially assess variable responses to levodopa treatment across PD patients. The previously established relationship between *tyrDC* presence and levodopa decarboxylation in PD patients demonstrates the direct contribution of *E. faecalis* in observed interindividual variability in treatment response [4]. While the abundance of *tyrDC* has been proposed as a predictive biomarker of levodopa metabolism in PD patient microbiotas, the complex nature of the gut makes VOCs a robust alternative for the non-invasive assessment of metabolic heterogeneity. Given that *tyrDC* abundance has been shown to positively correlate with levodopa dosages in PD patient samples [4], future studies also analyzing the abundance of 2,6-dimethylpyrazine and 4,6-dimethylpyrimidine from complex samples could explore their potential as indicative markers of levodopa metabolism.

We also detect a shift in the volatile metabolome of *C. sporogenes* during the bioconversion of levodopa to DHPPA that corresponds to an increased abundance of VOCs classified as fatty acid esters. These energy-rich compounds are associated with pathways of cell signaling, membrane formation, and fatty acid biosynthesis. Moreover, fatty acid esters are associated with the distinct metabolite profile detected from *C. sporogenes* during anaerobiosis, but not aerobiosis [36]. The production of short and medium chain fatty acids is well implicated across Clostridia species [37–39], with various metabolic pathways producing a variety of fatty acid products via chain elongation mechanisms using coenzyme A [37, 40]. Similarly, the fldABC protein complex responsible for DHPPA production uses the same CoA-dependent transfer mechanism to deaminate levodopa [5]. These processes generate an accumulation of ester precursors that enables their production. Although we did not observe statistically significant differences in VOC abundances between *C. sporogenes* cultured with and without levodopa, we report previously uncharacterized compounds that reflect the strain’s unique metabolic activity. While these VOCs have been implicated in cellular processes underlying energy production, further investigations are needed to link the underlying biological pathways leading to their production.

We next sought to characterize VOCs associated with the dehydroxylation of the levodopa intermediate DHPPA. *E. lenta* performs catechol dehydroxylation using distinct molybdenum-dependent enzymes that are variably distributed across individual strains [4, 23]. The prevalence of genes encoding these specialized enzymes correlates with the metabolism of their specific substrates, with hcdh activity being the most prevalent across closely related gut microbiota [23, 41]. As such, there is a growing interest to elucidate the chemical mechanisms exerted by these enzymes to mediate primary and secondary metabolism in the gut. To better understand the effects of catechol dehydroxylation, we identified significant changes in VOCs detected during DHPPA metabolism by hcdh. During the dehydroxylation of DHPPA to 3-HPPA, we detect a significant increase in 4,5-dimethylpyrazine and a putative dimethylpyrimidine isomer from *E. lenta*. With the mechanisms of catechol dehydroxylase enzymes remaining poorly understood, it is difficult to decipher their biological roles and pathway involvements. However, additional work from Rekdal et al. demonstrated that the dehydroxylation of dopamine, the other levodopa intermediate, provided a growth advantage to *E. lenta* and potentially serves as an alternative electron acceptor [4, 23]. Furthermore, metabolite profiles recently generated by Noecker et al. revealed that nucleotide and cell wall metabolites comprised a large proportion of the *E. lenta* metabolome, along with nucleic acid intermediates [42]. Based on these findings, we suspect the production of 4,5-dimethylpyrimdine and the other DHPPA-associated VOC could be a result of the potential growth-promoting effects of DHPPA metabolism. These studies also show that the addition of tungstate to the growth medium blocks the molybdenum-dependent dehydroxylase activity and inhibits the growth increase of *E. lenta* [4]. Interestingly, supplementing tungstate to *E. lenta* cultures suppressed the production of 4,5-dimethylpyrimidine and the putative dimethylpyrimidine isomer, providing evidence for endogenous production and further suggesting these VOCs may arise from increased growth tied to catechol dehydroxylation. While further investigations are necessary to characterize the dimethylpyrimidine isomer and confirm the biological origins of these VOCs, previous works have demonstrated the role of pyrimidines and their derivatives for bacterial growth and sensing mechanisms, implicating metabolic pathways for cell growth and signal processing as potential sources for VOCs [43–45]. Taken together, these findings open the door to improve our understanding of the relationship of dimethylpyrimidine VOCs and bacteria growth to improve our understanding of the effects of catechol dehydroxylation on gut microbiota metabolism.

VOCs can be generated across a wide range of biosynthetic pathways and are thought to diffuse through cell membranes- giving them the potential to modulate gene expression and influence physiology [28]. Thus, an emerging area of research is focused on understanding the molecular mechanisms underlying VOC production. While little information is known regarding the genes and pathways that interact with VOCs, the main biological responses reported for microbial VOCs include biofilm formation, virulence, secondary metabolite production, and growth [28]. In line with these findings, many of the VOCs identified in this study are implicated in those cellular processes. In this study, we detected unique volatile signatures from *E. faecalis*, *C.sporogenes*, and *E. lenta*. Functional group analyses revealed diverse chemical compositions within the VOC profile of each strain, whose relative abundances reflect different biological processes. The VOC profile of *E. faecalis* was mainly composed of heteroaromatic compounds, followed closely by aldehydes and VOCs classified as “other.” Additionally, we identified analyte 1 from *E. faecalis* as an endogenous VOC. We suspect that these compounds originate from primary metabolic pathways encoded in lactic acid bacteria and are likely products of carbohydrate fermentation and amino acid degradation. The VOC profile of *E. lenta* showed a similar chemical composition after performing functional group analyses. Little is known about the metabolic activity of *E. lenta* and the biosynthetic pathways leading to VOC production. Interestingly, the VOCs we detected from *E. lenta* are implicated in processes for amino acid and nucleic acid metabolism. These findings are in agreement with previous studies using systems biology-based approaches, which show that a large portion of *E. lenta’s* metabolic activity corresponds to cell growth and energy metabolism [41]. Collectively, these findings support that the distinct volatile profiles capture differences in metabolic activity across bacteria strains. Furthermore, the chemical signals encoded in these signatures can elicit a range of processes to coordinate behavior and respond to environmental stimuli. Thus, it is likely that the organism-specific VOC profiles detected in this study have distinct biological effects.

Here, we report differences in the VOC profiles of *E. faecalis*, *C. sporogenes*, and *E. lenta* and identify VOCs associated with levodopa metabolism. Using an untargeted, metabolomics approach, we report previously uncharacterized VOCs from each strain that furthers our understanding of metabolic processes harnessed by different gut bacteria. Collectively, these findings give insight into the metabolic activities of different gut microbiota, identify reproducible changes that occur during the breakdown of levodopa, and link 4,6-dimethylpyrimidine and 2,6-dimethylpyrazine to the endogenous bioconversion of levodopa by *E. faecalis*. Our consideration of species-specific VOCs shows diverse chemical classes within each organism’s volatilome that reflect interspecies differences in metabolic activity. The findings in this work open the door for further, targeted analyses of discriminant VOCs of levodopa metabolism and further exploration of their biological significance. In the future, functional interpretations of these VOCs will enable us to decode the molecular mechanisms by which gut bacteria interfere with PD treatment, offering novel strategies to assess treatment efficacy and improve patient outcomes.

## Methods

### Bacterial growth and incubation

All bacteria culturing work was performed in a glove bag (Sigma-Aldrich, SKU #Z530212) under an atmosphere of 5% H_2_, 20% CO_2_, and 80% N_2_. Plates were sealed in an anaerobic jar (Thomas No. 1177 × 01) containing an Anaerobe GasPak satchet (BD, SKU #260,678) and incubated at 37 ℃. Hungate tubes (Chemglass, CLS-4208) were used for all liquid culturing and incubated at 37 ℃ in a tube shaker at 500 rpm.

*Enterococcus faecalis OG1RF* (ATCC 47077) and *Clostridium sporogenes* (ATCC 15579) were plated onto Brain Heart Infusion (BHI) agar (Sigma-Aldrich, SKU #70138-500G) and incubated for 24 and 48 h, respectively. Isolated colonies were then inoculated into BHI broth (Sigma-Aldrich, SKU #53286-100G) and grown 24 h for *E. faecali*s, and 48 h for *C. sporogenes*. *Eggerthella lenta* DSM 15644 *(*ATCC 43055) was plated onto BHI agar containing 1% arginine (Sigma-Aldrich, #A5006-100G) and incubated for 48–72 h. Isolated colonies were then inoculated into BHI broth containing 1% arginine and 10 mM sodium formate (Sigma-Aldrich, #71539-500G) and grown for 48 h.

### VOC collection

OD_600_ measurements were taken and used to dilute turbid starter cultures such that the initial OD_600_ seeding concentration was approximately 0.1 at the start of VOC collection and subsequently used for statistical normalization. To evaluate the bioconversions of levodopa by *E. faecalis* and *C. sporogenes*, bacteria were seeded into the custom glass culture vessel containing a total 5 mL broth with and without 1 mM levodopa (Sigma-Aldrich, SKU #D9628-5G). To evaluate dehydroxylation of the intermediate metabolite DHPPA by *E. lenta*, bacteria were seeded into the culture vessel containing 5 mL of broth with and without 500 µM DHPPA (Sigma-Aldrich, SKU #102601-2.5G).

Bacteria cultures were then connected to a custom flow system designed to collect VOCs from each bacteria strain using dynamic headspace sampling methodology (Fig.S1). Anaerobic gas (5% H_2_, 20% CO_2_, 80% N_2_) was connected to the custom glass culture vessel inlet using Nalgene™ 890 FEP tubing in-line with a hydrocarbon trap, sterile filter, and flow meter. The gas flow was turned on 24 h prior to all VOC collection in order to obtain equilibrium and maintain a flow rate of 18 mL/min for the duration of sampling. The flow rate was lowered to 8.6 mL/min for *E. lenta* due to the longer 48 h sampling duration. A DVB/Carboxen/PDMS SPME fiber (Sigma-Aldrich, SKU #57298-U) was used to recover VOCs from our in-house system[17]. The SPME fiber was connected to the system using a previously described adapter component [17] attached directly to the outlet of the custom glass culture vessel. Each SPME fiber was replaced after approximately every 80 injections and conditioned at 270 ℃ prior to use. Fibers were heated at 270 ℃ for 5 min between sample injections to minimize carryover.

The sampling duration for each strain was determined based on preliminary data determining the metabolic rates of levodopa or DHPPA by the respective strains [4, 5]. To extract VOCs, the SPME fiber was exposed in the headspace of *E. faecalis* cultures for 10 h, *C. sporogenes* cultures for 24 h, and *E. lenta* cultures for 48 h. All experiments were done in triplicate for each experimental group. Media controls were collected in triplicate following the same sampling procedures for each strain. Control replicates only contained BHI broth and were not inoculated with bacteria in order to account for exogenous VOCs produced from either the culture medium or analytical system.

### Gas chromatography-mass spectrometry analysis

VOCs were analyzed using an Agilent 6890 GC-MS and an HP-5MS column (30.0 m x 250 μm x 0.25 μm; Agilent #19091S-433). SPME fibers were injected into the inlet using a Gerstel MPS autosampler maintained at 250 ℃, using splitless injection. The column was heated to an initial temperature of 60 ℃, ramped at 5 ℃/min to 100 ℃, then ramped at 30 ℃/min to 270 ℃ and held for 2 min. The helium carrier gas flow rate was 1.0 mL/min (UHP Helium 99.999%). The transfer line was maintained at 280 ℃. For mass spectrometry, the source temperature was 230 ℃. Mass spectra were acquired over a mass range of 35–300 amu with an ionization energy of -70 mV. A PFTBA standard was run prior to each SPME injection to tune the MS in an effort to minimize instrument variability. A 1 µL volume of methanol was injected prior to each sample to monitor for contaminants and further minimize carryover.

### Data processing

MSD ChemStation software was used to analyze the chromatograms from each run. Quantitative values for signal abundance were obtained by integrating the area under each peak in the chromatogram. Full-width half maximum was used to define the peak integration parameters. Each peak was compared to the Wiley and National Institute of Standards and Technology (NIST) 2005 Mass Spectral Libraries, and tentative peak names were assigned using reference mass spectra for each compound if the spectral similarity was ≥ 70 (70%). All integrated peaks and library hits were exported into Excel and the dataset for each replicate was saved as a .csv file.

Each dataset was imported into R software, version 4.1.2 (The R Foundation for Statistical Computing, Vienna, Austria). Custom R code was designed to streamline post-processing, statistical analysis, and data visualization. Peaks were integrated using the total ion chromatogram and filtered using a minimum threshold value of 1,000,000 for the integrated abundance to focus on the reproducible features in each bacterial volatilome. 

To align peaks across chromatograms, the retention time shift had to be ≤ 0.6 s between samples. Compound abundance was normalized using the OD_600_ measurement recorded at the start of each replicate. The resulting dataset was then manually filtered to remove known contaminants (siloxanes) and poorly resolved peaks.

VOC identities were assigned according to the metabolomic reporting standards set previously [46]. Compounds received an ID confidence level between 1 and 4, with 1 representing the highest confidence supported by two independent and orthogonal data sources [46]. In brief, compounds with mass spectral match ≥ 80%, to the Wiley and NIST 2005 Mass Spectral Libraries, received an initial confidence level of 3. Compounds below this mass spectral threshold were assigned an ID level of 4 and labeled as numbered analytes (i.e. “Analyte 1”). A C8-C20 alkane standard was analyzed and used to assign retention indices (RI) for all VOCs. Compounds with an ID level of 2 were verified with a ≥ 80% mass spectral match and a retention index that is consistent with the non-polar stationary phase using the mean of published RIs. Analytical standards (≥ 98%) were characterized under identical conditions (Fig. S2) and were used to assign an ID level of 1 (TCI #D1527; TCI #D2171).

### Statistical analysis

Compounds variably present (> 30% missing observations) in each dataset were removed, unless otherwise indicated, to focus on the reproducible aspects of the bacterial volatile metabolome. Integrated peaks were log_10_ transformed in R, Version 4.0.3 (The R Foundation for Statistical Computing, Vienna, Austria) and missing values were imputed to 0 abundance. A two-sided, unpaired students’ t-test assuming unequal variance was applied to compare VOC abundances between each experimental condition in a pairwise manner. The resulting *p*-values were then adjusted according to the Benjamini-Hochberg correction procedure [47] using the “p.adjust” function in the base R “stats” library.

### Enzyme inhibition

To determine whether VOCs significant to levodopa metabolism were produced as a direct result of its degradation, bacteria cultures were treated with known inhibitors of levodopa metabolism [4]. *E. faecalis* cultures were supplemented with 1 mM levodopa and 250 µM (S)-𝛼-fluormethyltyrosine (AFMT) (Sigma Aldrich, SKU # SML3100-5MG) using the same procedures described previously to prevent levodopa decarboxylation by the tyrDC enzyme. To inhibit DHPPA dehydroxylation by *E. lenta*, 250 µM sodium tungstate (Sigma Aldrich, SKU # 72069-25G) was added to *E. lenta* cultures containing 500 µM DHPPA. Three experimental replicates were collected from each strain using the same procedures described previously to determine the resulting effects on VOCs identified as significant. All inhibition studies were performed in experimental triplicate using the methodology described previously.

### Electronic supplementary material

Below is the link to the electronic supplementary material.

Supplementary Material 1

## Data Availability

The datasets generated and analyzed during the current study are available in the FigShare repository [48], 10.6084/m9.figshare.25769043, and are being evaluated at the MetaboLights [49] repository under MTBLS8293.

## Code availability

Custom R code designed to streamline post-processing, normalization, statistical analysis, and data visualization has been made publicly available in the Smith Lab Github repository (https://github.com/BSmithLab/MicrobiomeMetaboliteAnalysis).
