# Supplementary material for: Identification of volatile metabolites produced from levodopa metabolism by different bacteria strains of the gut microbiome
Source: BMC Microbiol. 2024 Jul 13;24:260. doi: 10.1186/s12866-024-03373-7 (PMC11245815; doi:10.1186/s12866-024-03373-7)
Supplement: Supplementary file 1 — Supplementary Material 1 [file 12866_2024_3373_MOESM1_ESM.pdf]

Supplementary Information for

Identification of volatile metabolites produced from levodopa metabolism by different bacteria strains of the gut microbiome

Taylor Pennington, Jarrett Eshima, Barbara S. Smith

Correspondence to: BarbaraSmith@asu.edu

This PDF file includes:

Figs. S1 to S2

Table S1 to S3

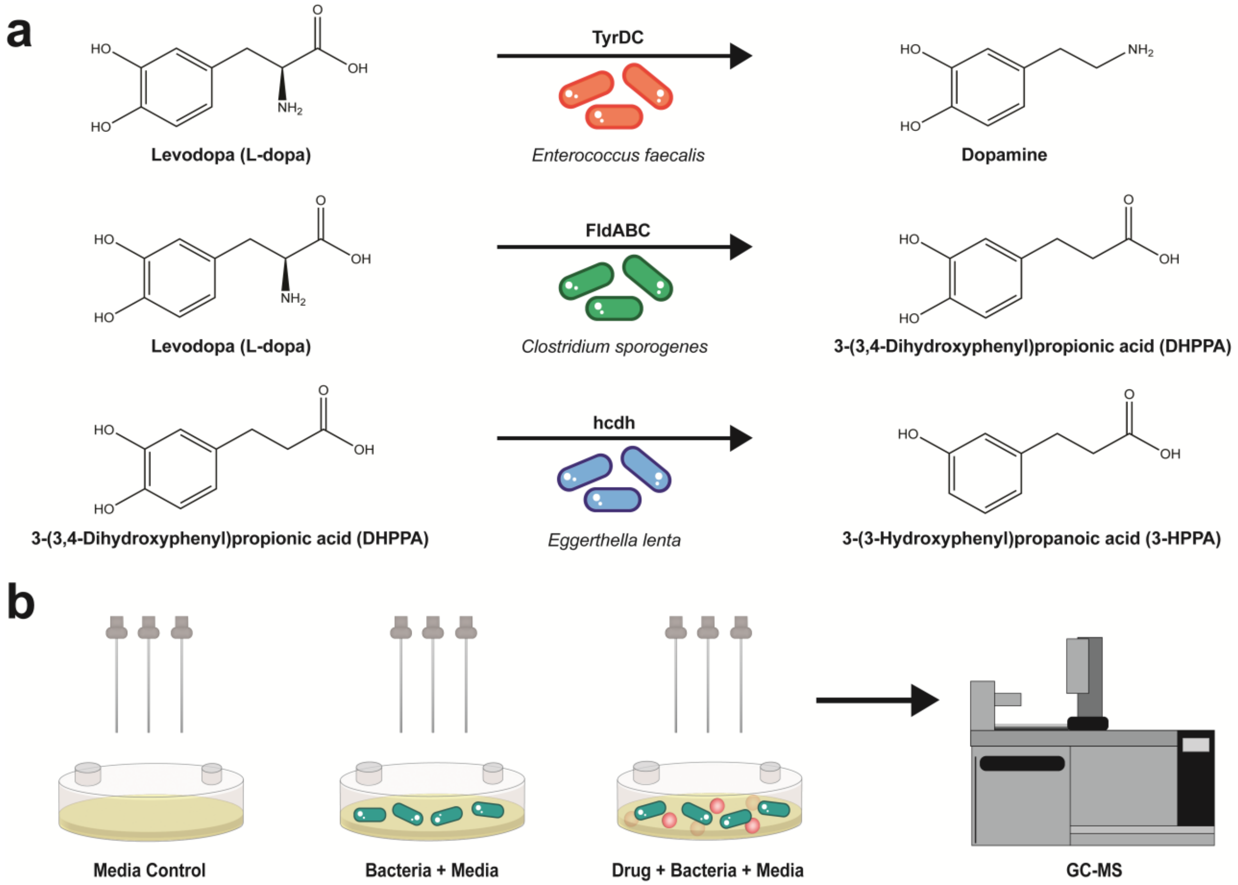

Fig. S1. Workflow implemented to identify volatile metabolites of gut microbial levodopa metabolism. (a) Reaction schemes depicting the biotransformation of levodopa and its resulting metabolites. *E. faecalis* tyrDC metabolizes levodopa to dopamine (top); *C. sporogenes* deaminates levodopa to DHPPA via the fldABC protein complex (middle); *E. lenta* dehydroxylates DHPPA to 3-HPPA via hcdh (bottom). **(b)** Diagram showing experimental setup to collect VOCs from *E. faecalis*, *C. sporogenes*, and *E. lenta* to analyze compounds produced during the breakdown of levodopa. Three replicates were acquired for each experimental group shown in (b).

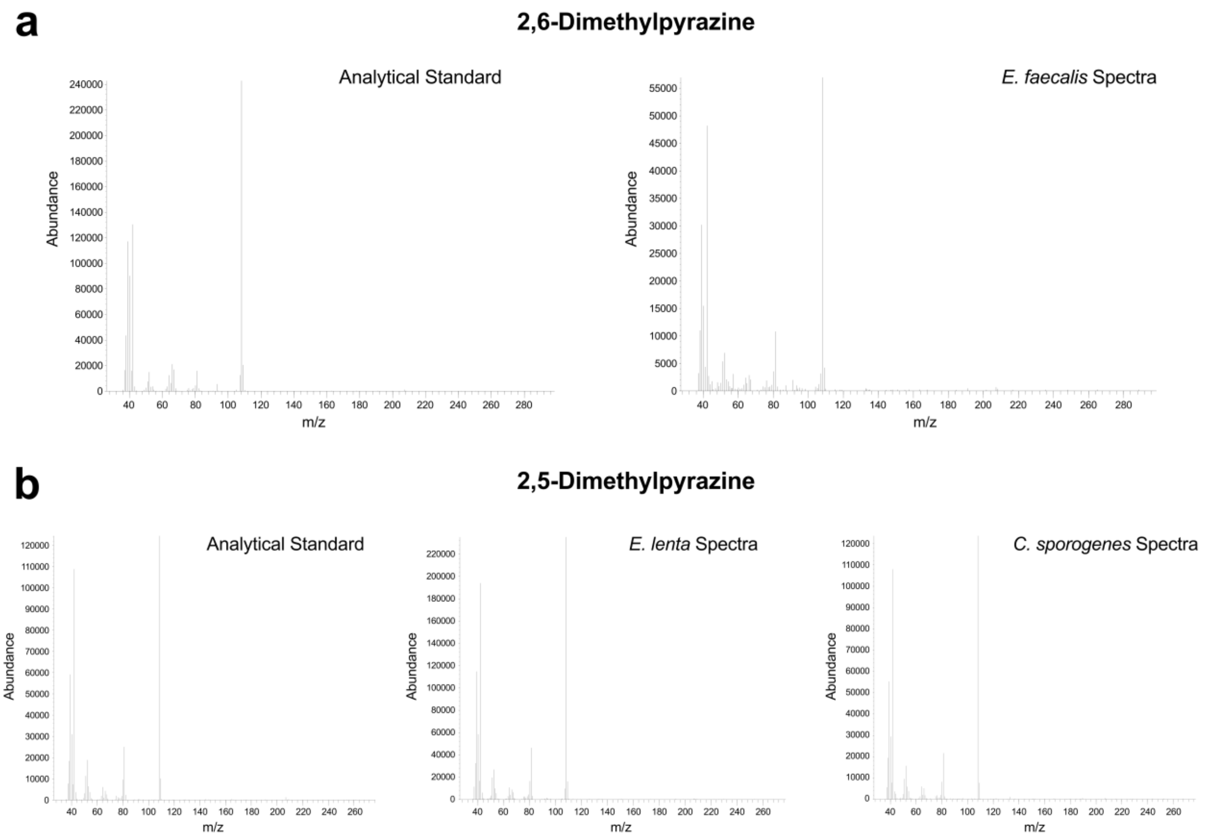

Fig. S2. Mass spectral identification of dimethylpyrazines with purified standards. (a) Mass spectra acquired from 2,6-dimethylpyrazine analytical standard (TCI) (left) and *in vitro* from *E. faecalis* (right). **(b)** Mass spectra acquired from 2,5-dimethylpyrazine analytical standard (TCI) (left), and *in vitro* from *E. lenta* (center) and *C. sporogenes* (right).

Table S1. *E. faecalis* statistical results.

Filtered VOC list from E. faecalis	*p-value*						*FDR adjusted p-value*					
**VOC name**	***Pos vs Neg***	***Pos vs Media***	***Neg vs Media***	***Pos vs Inhib***	***Neg vs Inhib***	***Media vs Inhib***	***Pos vs Neg***	***Pos vs Control***	***Neg vs Control***	***Pos vs Inhib***	***Neg vs Inhib***	***Media vs Inhib***
sec-Butylamine	0.29596	0.21278	0.02131	0.65132	0.00325	0.00947	0.52136	0.43073	0.09031	0.75282	0.01699	0.04448
Analyte 1	0.51059	0.00021	0.35507	0.00049	0.95812	0.00006	0.66827	0.00309	0.53362	0.00318	0.98014	0.00176
Methylpyrazine	0.04621	0.23194	0.55922	0.55796	0.08492	0.35974	0.15231	0.44875	0.68179	0.68179	0.24379	0.53362
o-Xylene	0.18351	0.54703	0.39935	0.42265	0.00013	0.19440	0.41807	0.68179	0.57326	0.58003	0.00232	0.42199
2,5-Dimethylpyrazine	0.62747	0.62489	0.46992	0.47403	0.35183	0.97792	0.74459	0.74459	0.62968	0.62968	0.53362	0.98904
2,6-Dimethylpyrazine	0.00057	0.00057	0.00057	NA	NA	NA	0.00318	0.00318	0.00318	NA	NA	NA
4,6-Dimethylpyrimidine	0.00051	0.00051	0.00051	NA	NA	NA	0.00318	0.00318	0.00318	NA	NA	NA
Benzaldehyde	0.13557	0.30540	0.64031	0.39273	0.32156	0.70712	0.33516	0.52270	0.74984	0.57300	0.52998	0.80684
Benzeneacetaldehyde	0.22037	0.55782	0.82731	0.14791	0.25334	0.52339	0.43584	0.68179	0.87655	0.34643	0.46974	0.67510
Acetophenone	0.01365	0.06050	0.87736	0.09250	0.02256	0.13001	0.06076	0.18568	0.91865	0.25726	0.09126	0.33313
4-Hydroxy-3-methylbenzaldehyde	0.32000	0.42362	0.23757	0.35137	0.33924	0.75003	0.52998	0.58003	0.44987	0.53362	0.53362	0.83440
3-Methylbenzaldehyde	0.00035	0.13053	0.99303	0.42265	0.00010	0.13101	0.00318	0.33313	0.99303	0.58003	0.00224	0.33313
Phthalan	0.00056	0.00056	0.18790	NA	0.00002	0.00002	0.00318	0.00318	0.41807	NA	0.00073	0.00073
Dodecane	0.05153	0.77098	0.88794	0.33732	0.14323	0.73213	0.16380	0.84713	0.91892	0.53362	0.34453	0.82481
Tetradecane	0.82363	0.26861	0.04428	0.29876	0.00950	0.80418	0.87655	0.48789	0.15157	0.52136	0.04448	0.87283
Phenol, 2,4-bis(1,1-dimethylethyl)	0.06669	0.03205	0.21294	0.20887	0.02820	0.02947	0.19785	0.11412	0.43073	0.43073	0.10913	0.10928

The compound name is presented first, followed by the unadjusted and FDR-adjusted p-values, respectively. The *p*-values were calculated using a two-tailed student's t-test comparing VOC abundance in each experimental condition post-filtering, then adjusted using the FDR procedure in R. NA = *p*-value of 0, Pos = with levodopa, Neg = no additive, Media = media control, Inhib = with levodopa + AFMT.

Table S2. ***C. sporogenes*** **statistical results.**

Filtered VOC list from C. sporogenes	*p-value*			*FDR adjusted p-value*		
**VOC name**	***Pos vs Neg***	***Pos vs Media***	***Neg vs Media***	***Pos vs Neg***	***Pos vs Media***	***Neg vs Media***
Analyte 1	0.52613	0.12604	0.16494	0.64073	0.30398	0.35593
Analyte 2	0.00530	0.18354	0.32781	0.02036	0.35833	0.53606
Analyte 3	0.61680	0.94754	0.65100	0.70247	0.96321	0.72793
Analyte 4	0.56726	0.00031	0.00391	0.67090	0.00342	0.01605
Analyte 5	0.00481	0.00012	0.44962	0.01910	0.00227	0.57279
sec-Butylamine	0.57835	0.00205	0.03795	0.67749	0.00870	0.12766
Analyte 6	0.75646	0.00002	0.00060	0.82340	0.00186	0.00343
Analyte 7	0.59186	0.03810	0.13494	0.68036	0.12766	0.31918
4-Methyl-1-pentanol	0.36173	0.88980	0.38063	0.53606	0.91971	0.53974
2,5-Dimethylpyrazine	0.65866	0.63997	0.88619	0.72986	0.72217	0.91971
Benzaldehyde	0.26972	0.04321	0.18107	0.48081	0.13627	0.35833
Butanoic acid, butyl ester	0.80226	0.29625	0.33468	0.85481	0.50609	0.53606
Trimethylpyrazine	0.80837	0.28849	0.42997	0.85481	0.49978	0.55669
Butyl 2-methylbutanoate	0.38177	0.00054	0.18350	0.53974	0.00343	0.35833
Butanoic acid, 3-methyl-, butyl ester	0.35365	0.11689	0.05660	0.53606	0.29341	0.16980
Pentanoic acid, butyl ester	0.38894	0.12248	0.36127	0.54363	0.30129	0.53606
Acetophenone	0.42463	0.33463	0.09265	0.55669	0.53606	0.24248
Butanoic acid, pentyl ester	0.98661	0.07596	0.05491	0.98661	0.21234	0.16886
Butanoic acid, 2-methyl-, 3-methylbutyl ester	0.10498	0.15045	0.15934	0.26900	0.34915	0.35593
Butanoic acid, 3-methyl-, 3-methylbutyl ester	0.78411	0.02641	0.04127	0.84602	0.09845	0.13359
n-Amyl isovalerate	0.40313	0.17138	0.51128	0.55669	0.35728	0.62888
Phenylethyl alcohol	0.25318	0.00013	0.00005	0.46479	0.00227	0.00186
Benzyl nitrile	0.98065	0.42923	0.42997	0.98661	0.55669	0.55669
Hexanoic acid, 3-methylpropyl ester	0.27475	0.00028	0.00004	0.48277	0.00340	0.00186
Dodecane	0.93316	0.58421	0.54855	0.95648	0.67790	0.65506
Hexyl n-valerate	0.30692	0.00053	0.00017	0.51715	0.00343	0.00261
Isopentyl hexanoate	0.48295	0.00061	0.00011	0.60615	0.00343	0.00227
Benzenepropanol	0.35015	0.00061	0.00066	0.53606	0.00343	0.00343
Hexanoic acid, pentyl ester	0.41996	0.00061	0.18351	0.55669	0.00343	0.35833
Analyte 8	0.41568	0.00077	0.00012	0.55669	0.00343	0.00227
Analyte 9	0.81311	0.16033	0.16255	0.85481	0.35593	0.35593
Tetradecane	0.34980	0.06664	0.03840	0.53606	0.19516	0.12766
Phenethyl butyrate	0.37325	0.00075	0.00034	0.53974	0.00343	0.00343
Benzeneacetic acid, butyl ester	0.24653	0.00078	0.00021	0.45944	0.00343	0.00290
Butanoic acid, 3-methyl-, 2-phenylethyl ester	0.24455	0.24162	0.26694	0.45944	0.45944	0.48081
Phenol, 2-4-bis(1,1-dimethylethyl)	0.45171	0.03390	0.06849	0.57279	0.12265	0.19591
Analyte 10	0.53978	0.37237	0.35854	0.65092	0.53974	0.53606
2-Methylpropyl benzenepropanoate	0.34149	0.00065	0.00056	0.53606	0.00343	0.00343
Butanoic acid, 3-phenyl propyl ester	0.73126	0.00037	0.00070	0.80308	0.00343	0.00343
Hexadecane	0.50553	0.08888	0.08318	0.62808	0.23766	0.22736
Pivalic acid, 2-phenylethyl ester	0.16986	0.00055	0.00077	0.35728	0.00343	0.00343

The compound name is presented first, followed by the unadjusted and FDR-adjusted p-values, respectively. The *p*-values were calculated using a two-tailed student's t-test comparing VOC abundance in each experimental condition post-filtering, then adjusted using the FDR procedure in R. Pos = with levodopa, Neg = no additive, Media = media control.

Table S3. *E. lenta* statistical results.

Filtered VOC list from E. lenta	*p-value*						*FDR adjusted p-value*					
**VOC name**	***Pos vs Neg***	***Pos vs Media***	***Neg vs Media***	***Pos vs Inhib***	***Neg vs Inhib***	***Media vs Inhib***	***Pos vs Neg***	***Pos vs Control***	***Neg vs Control***	***Pos vs Inhib***	***Neg vs Inhib***	***Media vs Inhib***
Methylpyrazine	0.03544	0.05296	0.00797	0.40639	0.00242	0.00275	0.17009	0.19258	0.05316	0.57803	0.01940	0.02060
2,5-Dimethylpyrazine	0.13116	0.60827	0.04857	0.18618	0.00933	0.02483	0.33013	0.74482	0.18443	0.39195	0.05891	0.13544
4,5-Dimethylpyrimidine	0.00011	0.00011	0.00011	NA	NA	NA	0.00260	0.00260	0.00260	NA	NA	NA
Analyte 1	0.00061	0.00061	0.00061	NA	NA	NA	0.00721	0.00721	0.00721	NA	NA	NA
Benzaldehyde	0.10704	0.33486	0.78660	0.23709	0.11873	0.01344	0.30464	0.53734	0.85757	0.44455	0.32380	0.08064
Trimethylpyrazine	0.13305	0.26218	0.10780	0.33584	0.03353	0.01411	0.33013	0.48372	0.30464	0.53734	0.16767	0.08064
Benzene, 1-methyl-2-(1-methylethyl)	0.53834	0.00063	0.67584	0.00350	0.79325	0.00220	0.67293	0.00721	0.77266	0.02468	0.85757	0.01886
Benzeneacetaldehyde	0.00076	0.13480	0.78237	0.00066	0.00019	0.07770	0.00759	0.33013	0.85757	0.00721	0.00382	0.25200
Acetophenone	0.20369	0.32127	0.16661	0.13234	0.08596	0.45794	0.40719	0.52812	0.38448	0.33013	0.26450	0.61059
3-Ethyl-2,5-dimethylpyrazine	0.18263	0.37928	0.08206	0.34570	0.04775	0.00154	0.39195	0.56190	0.25914	0.54585	0.18443	0.01419
3-Methylbenzaldehyde	0.04226	0.04632	0.88824	0.74872	0.06250	0.07606	0.18443	0.18443	0.92366	0.83968	0.22058	0.25200
Nonanal	0.23348	0.81245	0.67608	0.20507	0.20699	0.80529	0.44455	0.86278	0.77266	0.40719	0.40719	0.86278
Benzyl nitrile	0.47819	0.96169	0.98717	0.51931	0.46528	0.94896	0.61702	0.97798	0.98717	0.66294	0.61356	0.97329
Dodecane	0.15017	0.09009	0.56457	0.63868	0.52515	0.69591	0.35954	0.27028	0.69843	0.75473	0.66335	0.78783
2-Methoxy-4-methylphenol	0.20592	0.44467	0.42382	0.37701	0.36467	0.98376	0.40719	0.59956	0.58192	0.56190	0.56190	0.98717
Nonanoic acid	0.07323	0.30771	0.47152	0.04317	0.04334	0.61919	0.25109	0.52812	0.61503	0.18443	0.18443	0.75053
Indole	0.02908	0.10916	0.62990	0.17175	0.04918	0.15280	0.15175	0.30464	0.75473	0.38886	0.18443	0.35954
Indolizine	0.00009	0.40944	0.67380	0.18352	0.00006	0.40325	0.00260	0.57803	0.77266	0.39195	0.00260	0.57803
Benzene, 3-cyclohexen-1-yl	0.31971	0.42674	0.82711	0.36819	0.40279	0.41712	0.52812	0.58192	0.87064	0.56190	0.57803	0.58192
Tetradecane	0.26605	0.31970	0.89287	0.12231	0.31288	0.30795	0.48372	0.52812	0.92366	0.32616	0.52812	0.52812
Phenol, 2-4-bis(1,1-dimethylethyl)	0.27963	0.37453	0.64152	0.18581	0.21667	0.77981	0.50082	0.56190	0.75473	0.39195	0.41935	0.85757

The compound name is presented first, followed by the unadjusted and FDR-adjusted p-values, respectively. The *p*-values were calculated using a two-tailed student's t-test comparing VOC abundance in each experimental condition post-filtering, then adjusted using the FDR procedure in R. NA = *p*-value of 0, Pos = with levodopa, Neg = no additive, Media = media control, Inhib = with levodopa + sodium tungstate.
